# Cortical and Subcortical Structural Plasticity Associated with the Glioma Volumes in Patients with Cerebral Gliomas Revealed by Surface-Based Morphometry

**DOI:** 10.3389/fneur.2017.00266

**Published:** 2017-06-09

**Authors:** Jinping Xu, Ahmed Elazab, Jinhua Liang, Fucang Jia, Huimin Zheng, Weimin Wang, Limin Wang, Qingmao Hu

**Affiliations:** ^1^Institute of Biomedical and Health Engineering, Shenzhen Institutes of Advanced Technology, Chinese Academy of Sciences, Shenzhen, China; ^2^Misr Higher Institute for Commerce and Computers, Mansoura, Egypt; ^3^Neurosurgery, Guangzhou General Hospital of Guangzhou Military Command, Guangzhou, China; ^4^Psychological Department, Guangzhou First People’s Hospital, Guangzhou, China

**Keywords:** structural plasticity, cerebral gliomas, gray matter volume, glioma volumes, surface-based morphometry

## Abstract

Postlesional plasticity has been identified in patients with cerebral gliomas by inducing a large functional reshaping of brain networks. Although numerous non-invasive functional neuroimaging methods have extensively investigated the mechanisms of this functional redistribution in patients with cerebral gliomas, little effort has been made to investigate the structural plasticity of cortical and subcortical structures associated with the glioma volume. In this study, we aimed to investigate whether the contralateral cortical and subcortical structures are able to actively reorganize by themselves in these patients. The compensation mechanism following contralateral cortical and subcortical structural plasticity is considered. We adopted the surface-based morphometry to investigate the difference of cortical and subcortical gray matter (GM) volumes in a cohort of 14 healthy controls and 13 patients with left-hemisphere cerebral gliomas [including 1 patients with World Health Organization (WHO I), 8 WHO II, and 4 WHO III]. The glioma volume ranges from 5.1633 to 208.165 cm^2^. Compared to healthy controls, we found significantly increased GM volume of the right cuneus and the left thalamus, as well as a trend toward enlargement in the right globus pallidus in patients with cerebral gliomas. Moreover, the GM volumes of these regions were positively correlated with the glioma volumes of the patients. These results provide evidence of cortical and subcortical enlargement, suggesting the usefulness of surface-based morphometry to investigate the structural plasticity. Moreover, the structural plasticity might be acted as the compensation mechanism to better fulfill its functions in patients with cerebral gliomas as the gliomas get larger.

## Introduction

Brain plasticity, defined as the continuous processing allowing short-, middle-, and long-term remodeling of the neurono-synaptic organization (1), has been described as natural plasticity for normal people in learning or memory (2), or postlesional plasticity for patients with traumatic brain injury, stroke, or tumors (3). In particular, more progressive lesions, such as cerebral gliomas, have been identified to induce a large functional reshaping of brain networks around the tumor (4), within the lesioned hemisphere (5) or in parts of the contralateral hemisphere homologous to the structures invaded (6–9), which were thought of as functional compensations (i.e., the absence of deficit). Moreover, these functional reorganizations were utilized to explain why slow infiltrative low-grade gliomas within the so-called “eloquent” areas (such as Broca’s areas, Wernicke’s areas, and the supplementary motor area) usually do not induce detectable neurological deficits (10) or why remove these regions usually does not induce permanent deficits (11–14). For instance, studies have described that totally removing the supplementary motor area can recover in a few weeks (15–18) by recruiting the contralateral homologous regions (19), despite the occurrence of an immediate postsurgical transient supplementary motor area syndrome. For patients with cerebral gliomas, although numerous non-invasive techniques have analyzed almost exclusively mechanisms of functional redistribution, little effort has been made to investigate the structural plasticity of cortical and subcortical structures.

Recently, T1-weighted morphometric analysis has allowed the distinct exploration of cortical morphometry over the entire cortex (20). The precise measurement of gray matter (GM) volume has been adopted in healthy individuals and various neurological diseases (21–24), showing potentials to investigate structural plasticity in patients with cerebral gliomas. The Statistical Parametric Mapping (SPM)^1^ and FreeSurfer^2^ are two widely used methods to analyze the structural data. However, using surface geometry to do inter-subject comparisons of cortical brain areas (25), FreeSurfer analyzes GM volumes as structures as a whole without voxel-wise comparisons between individual magnetic resonance (MR) images, which exhibits higher sub-voxel accuracy than SPM based on voxel-based methods and is more robust to missegmentation (26). Therefore, investigating the structural morphometry of the cortical and subcortical structures with FreeSurfer could provide novel insights into the compensation mechanism in the patients with cerebral gliomas.

The goal of this research is to focus on the possible existence of a real cortical and subcortical plastic potential and try to answer whether the subcortical and contralateral cortical structures are able to actively reorganize by themselves in patients with cerebral gliomas. To this end, we adopted the surface-based morphometry to investigate the differences of cortical and subcortical GM volume in a cohort of 13 patients with cerebral gliomas and 14 healthy controls.

## Materials and Methods

### Participants

In this study, 13 patients with histologically confirmed cerebral gliomas were recruited consecutively from the Guangzhou General Hospital of Guangzhou Military Command. Of the 13 patients, 1 had World Health Organization (WHO) grade I, 8 were WHO II, and 4 were WHO III cerebral glioma (Table 1). Patient data were retrospectively obtained from our institutional database and had been acquired between 2013 and 2016. Patients were filtered to include only those with left frontal and/or temporo-parietal lobe brain gliomas. A small number of patients with right hemispheric gliomas were excluded from further analysis in order to provide a more homogeneous patient population. The glioma volume of each patient was calculated based on the preoperative T2 flair image using the OsiriX Lite (27–30)^3^ (Table 1). Fourteen healthy controls matched for sex and age were also recruited. All of the healthy controls underwent extensive neurologic, neuropsychologic, and clinical imaging examinations. Participants who had a history of neurologic or psychiatric disease and neurologic sequelae induced by brain trauma were excluded. All the patients and healthy controls are right handed. The study protocol was approved by the Guangzhou General Hospital of Guangzhou Military Command. Written informed consents were obtained from all participants after they received a complete description of the study. All procedures were performed according to the principles expressed in the Declaration of Helsinki.

**Table 1:** Demographic data in patients with cerebral gliomas.

Patients	Age (years)	Gender	Type of glioma	Glioma grades	Glioma locations	Glioma volume (cm^3^)
Patient 01	50	Male	Oligodendroglioma	WHO II	Left frontal cortex	71.1156
Patient 02	38	Male	Mixed^a^	WHO II	Left frontal cortex	10.0577
Patient 03	34	Male	Oligodendroglioma	WHO II	Left frontal cortex	31.2978
Patient 04	56	Male	Mixed	WHO III	Left frontal cortex	39.2518
Patient 05	46	Male	Ganglioglioma	WHO II	Left frontal cortex	38.4354
Patient 06	23	Female	Oligodendroglioma	WHO I	Left frontal cortex	5.1633
Patient 07	43	Male	Oligodendroglioma	WHO II	Left frontal cortex	88.5927
Patient 08	55	Female	Mixed	WHO II	Left frontal/temporal cortex	10.6836
Patient 09	17	Male	Anaplastic astrocytoma	WHO III	Left frontal cortex	208.185
Patient 10	20	Male	Astrocytoma	WHO II	Left frontal cortex	166.4146
Patient 11	25	Male	Anaplastic astrocytoma	WHO III	Left frontal cortex	83.7768
Patient 12	44	Female	Oligodendroglioma	WHO III	Left frontal cortex	44.3501
Patient 13	52	Male	Astrocytoma	WHO II	Left frontal cortex	71.1445

*WHO, World Health Organization*.

*^a^The type of glioma is mixed with oligodendroglioma and astrocytoma*.

### MRI Data Acquisition

MRI data were acquired on a 3.0-T MR imaging system (GE Medical Systems) in the Guangzhou General Hospital of Guangzhou Military Command. Whole brain structural images were acquired with a three dimensional (3D) T1-weighted 3D-bravo sequence. Detailed scan parameters were repetition time = 11.952 ms, echo time = 5.036 ms, inversion time = 380 ms, slice thickness = 1 mm, no gaps, flip angle = 15°, acquisition matrix = 256 × 256, and 0.47 mm × 0.47 mm in-plane resolution.

### Volumetric Analysis

Each scan was processed using the FreeSurfer pipeline, which is a semi-automated approach described in detail in prior publications (31, 32). Briefly, the image processing included skull stripping, automated Talairach transformations, segmentation of the subcortical white matter (WM) and deep GM structures, intensity normalization, tessellation of the boundary between GM and WM, automated topology correction, and surface deformation along intensity gradients for optimal placement of the borders between GM, WM, and cerebrospinal fluid. In case of inaccuracies, manual editing was performed according to the FreeSurfer editing manual,^4^ either by adding control points to help FreeSurfer identify the WM voxels or removing the skull and dura in case they were considered to be parts of the brain.

The GM volumes of subcortical structures including the bilateral thalamus, hippocampus, amygdala, putamen, globus pallidus, and caudate were calculated using the automated procedure for volumetric measurements of brain structures implemented in the FreeSurfer (33).

### Statistical Analysis

The cortical GM volume was analyzed using a surface-based group analysis of FreeSurfer’s Qdec (version 1.5). First, the spatial cortical GM volume of the right hemisphere was smoothed with a circularly symmetric Gaussian kernel of 10 mm full width half maximum to provide normal distribution of the results. Then, we employed a general linear model (GLM) analysis with age and gender as the nuisance factors in the design matrix to directly compare the GM volume in the right hemisphere of the two groups. Finally, the GLM result was corrected for multiple comparisons utilizing a pre-cached cluster-wise Monte Carlo simulation (34) implemented in Qdec (mc-z, threshold: *p* < 0.001, sign: absolute).

Volumes of the subcortical regions were measured automatically. Comparisons in the volumes of these regions were performed using the GLM analysis with age and gender as covariates using IBM SPSS 19.0 (IBM, Armonk, NY, USA).

Correlations between the glioma volumes of the patients and the GM volumes of the altered regions were analyzed with Pearson’s correlations in SPSS.

## Results

### Subject Characterization

There was no significant difference in terms of gender (patients: 10 males and 3 females, healthy controls: 9 males and 5 females, χ^2^ test, *p* = 0.678) and age (patients: 38.69 ± 13.68 years, healthy controls: 39.78 ± 15.12 years, two-sample *t*-test, *p* = 0.846) between patients with cerebral gliomas and healthy controls in the study.

### Difference of the GM Volume

**Figure 1:**
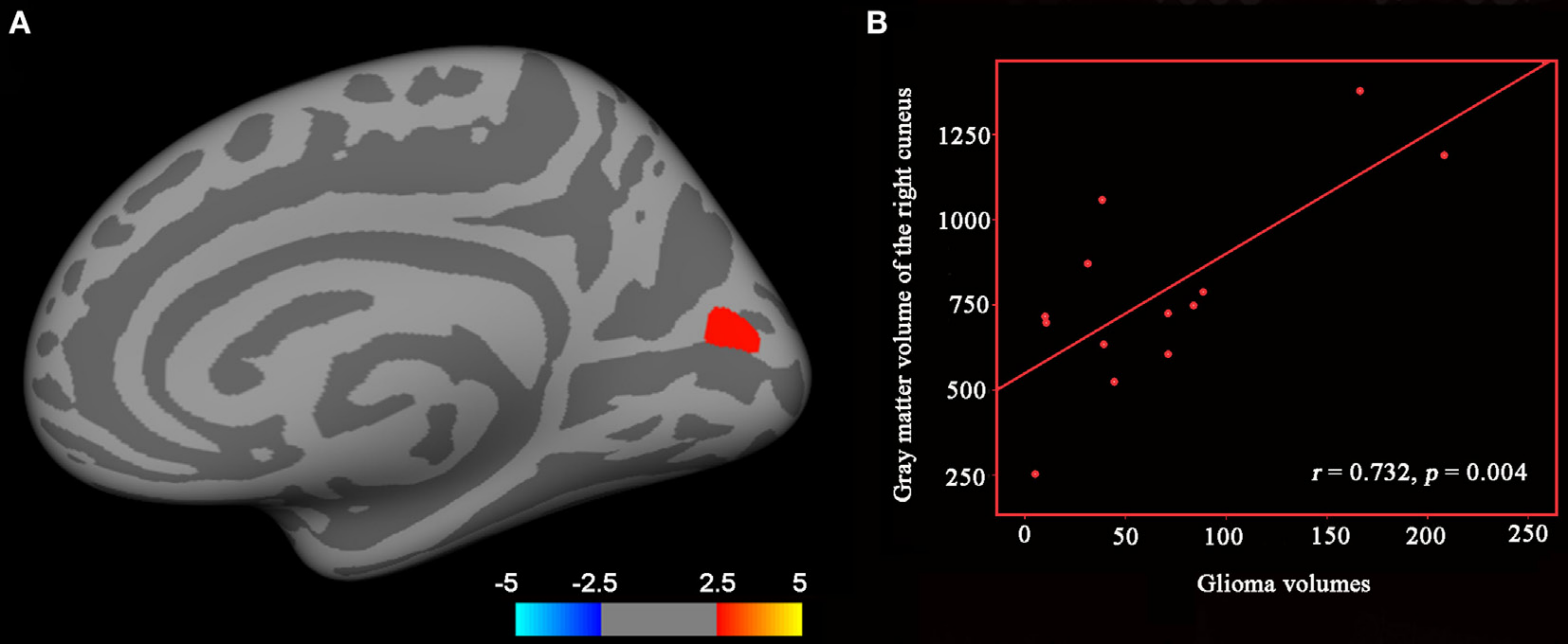
**(A)** Significantly increased gray matter (GM) volume of the right cuneus in patients with cerebral gliomas compared to healthy controls. Comparison was performed using the GLM analysis and corrected for multiple comparisons utilizing a pre-cached cluster-wise Monte Carlo simulation implemented in Qdec (mc-z, threshold: *p* < 0.001, sign: absolute). **(B)** Significantly positive correlation between glioma volumes and the GM volume of the right cuneus was identified in the patients with cerebral gliomas (*p* < 0.05).

**Figure 2:**
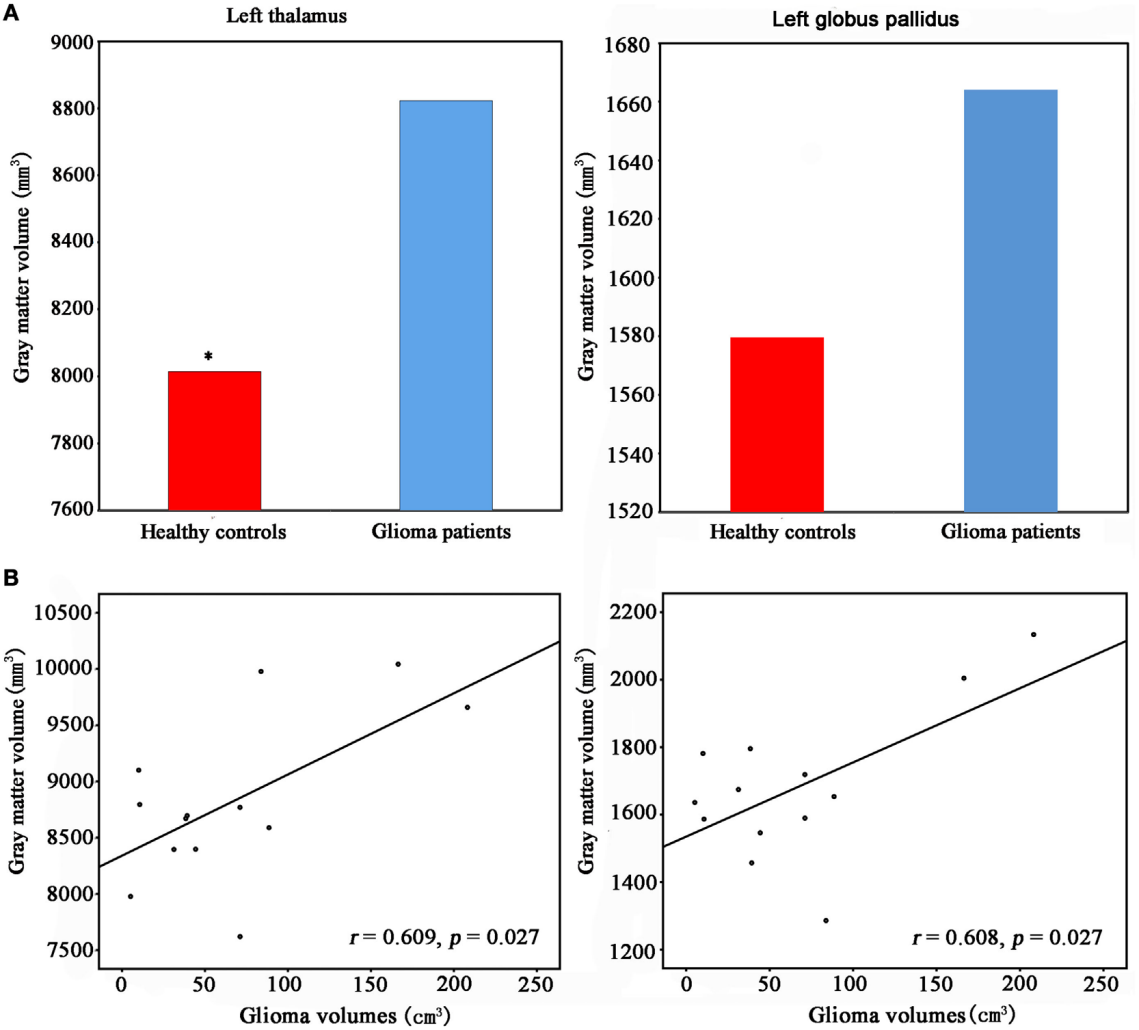
**(A)** Increased gray matter (GM) volume of the left thalamus and the left palladiums in patients with cerebral gliomas compared to healthy controls. Comparison was performed using the GLM analysis in IBM SPSS 19.0 (IBM, Armonk, NY, USA) with *p* < 0.05. *represents significant difference between the two groups. **(B)** Significantly positive correlation between glioma volumes and the GM volume of the left thalamus and palladiums were identified in the patients with cerebral gliomas (*p* < 0.05).

We found significantly increased GM volume in the right cuneus in patients with cerebral gliomas compared to healthy controls (Figure 1A). We also found significantly increased GM volume in the left thalamus and a trend toward enlargement in the right globus pallidus (Figure 2A; Table 2) in patients with cerebral gliomas.

**Table 2:** The comparisons of gray matter volumes in the subcortical regions between patients with cerebral gliomas and healthy controls.

Subcortical regions	Healthy controls Mean ± SD	Glioma patients Mean ± SD	*p* Value
Left thalamus	8,013.59 ± 831.65	8,822.00 ± 719.81	0.016
Left caudate	4,117.62 ± 682.02	3,819.09 ± 604.99	0.201
Left putamen	6,034.70 ± 1,044.38	5,861.19 ± 661.06	0.412
Left globus pallidus	1,579.74 ± 111.90	1,681.95 ± 219.50	0.196
Left hippocampus	4,091.42 ± 520.36	4,325.80 ± 524.25	0.341
Left amygdala	1,576.88 ± 274.69	1,634.13 ± 250.47	0.790
Right thalamus	7,126.80 ± 1,056.39	7,689.70 ± 1,090.49	0.231
Right caudate	3,910.25 ± 561.10	4,239.59 ± 551.22	0.200
Right putamen	5,811.27 ± 831.00	5,965.38 ± 686.83	0.802
Right globus pallidus	1,626.46 ± 181.70	1,770.34 ± 173.53	0.057
Right hippocampus	4,204.76 ± 647.72	4,480.26 ± 368.66	0.264
Right amygdala	1,610.53 ± 218.86	1,785.05 ± 258.81	0.104

### Correlations of the GM Volume and the Glioma Volumes of Patients

We found significantly positive correlations between the GM volume in the right cuneus and the glioma volumes in patients with cerebral gliomas (Figure 1B). We also found significantly positive correlations between GM volume in the left thalamus/the left globus pallidus and the glioma volumes in patients with cerebral gliomas (Figure 2B).

## Discussion

Using surface-based morphometric analysis, we found significantly increased GM volume in the right cuneus and the left thalamus, as well as a trend toward enlargement in the right globus pallidus in patients with cerebral gliomas compared to healthy controls. Moreover, the GM volumes of these regions were positively correlated with the glioma volumes of the patients.

Interestingly, we found significantly increased GM volume in the right cuneus and the left thalamus in the patients with cerebral gliomas, both of which were identified as hub regions in brain network. Functionally, the cuneus was identified as a hub region in a functional brain network (35), which connected to a visual network and acted as integrated center of the visual processing. Structurally, the cuneus was also identified as a hub region in the GM structural network and the diffusion-based network (36, 37). As for the thalamus, it has been identified as the a hub region in a brain network analysis and was believed to be a major processor of visual, auditory, and somatosensory information (35). Moreover, the thalamus is believed to act as a relay station by controlling the flow of sensorimotor information to and from the cortex (38, 39), especially in the cortical-striato-thalamic loop. Besides multiple functions, such as coordinating, encoding, retrieval, and planning (40), recent evidence supports a more diverse role of the thalamus in higher order cognitive functions by thalamocortical connections (41, 42). Given all these important parts the cuneus and the thalamus take in the brain functions, it is reasonable to speculate that the enlargement of these regions might be considered as the compensation mechanism to better fulfill its functions in patients with cerebral gliomas as the gliomas get larger. However, a previous review (10) suggested that mechanisms for plasticity are based on a hierarchically organized model involving three levels recruited successively. Our finding of GM volume enlargement in the hub regions might extend their suggestions to involve four levels. Moreover, this structural reorganization demonstrated that the subcortical and contralateral cortical structures are able to actively reorganize by themselves in patients with cerebral gliomas, which might be thought of as a novel explanation to why patients with gliomas usually do not have detectable neurological deficits. However, the mechanisms of brain plasticity for patients with cerebral gliomas were mostly based on functional MRI, which identified a large functional reshaping of brain networks (5–7). To the best of our knowledge, it is the first study that identified cortical and subcortical plasticity in patients with cerebral gliomas using surface-based morphometry. Therefore, our findings of larger GM volume in the cortical and subcortical regions were not hypothesized based on studies published to date and warrant replication.

Whether larger cortical and subcortical GM volume in patients with cerebral gliomas are resulted from the postlesional plasticity, or due to group differences in other confounding factors remain to be determined. Though given the wide range ages in the overall sample as well as a 17-year-old male in the patients group, there is no significant difference of age between the two groups. Moreover, we used GLM analysis with age as the covariate to minimize its impact. Other general confounding factors, such as differences in head motion during scanning would not likely uniquely affect the GM volume of the left cuneus and the left thalamus but would likely affect multiple other regions. Despite of the possibility of various confounding factors, our findings of cortical and subcortical enlargement might be related to the postlesional plasticity in patients with cerebral gliomas.

Some limitations should be stressed in this study. First, due to the loss of cognitive and behavior measurements of the patients, we were unable to address the relationship between these measurements and the GM volumes of the right cuneus/left thalamus, which weaken the interpretation of our finding. Second, our study was limited to patients with left cerebral glioma. Another subgroup of patients with right cerebral glioma is wanted to validate whether there is also a significant increase of GM volumes in the cortical and subcortical regions. Additionally, longitudinal and integrated structural–functional correlations might be necessary to further uncover the compensation mechanism for patients with cerebral gliomas.

To the best of our knowledge, it is the first study that identified cortical and subcortical plasticity in patients with cerebral gliomas, suggesting the usefulness of surface-based morphometry to investigate the structural plasticity. Moreover, the study might imply that the cortical and subcortical structures are able to actively reorganize by themselves and might be acted as the compensation mechanism to better fulfill its functions in patients with cerebral gliomas as the gliomas get larger. This structural reorganization might be thought of as a novel explanation to why patients with cerebral gliomas usually do not have detectable neurological deficits.

## Ethics Statement

The study protocol was approved by the Guangzhou General Hospital of Guangzhou Military Command. Written informed consents were obtained from all participants after they received a complete description of the study. All procedures were performed according to the principles expressed in the Declaration of Helsinki.

## Author Contributions

QH, FJ, and WW designed the research. LW and JL collected the data. JX and AE analyzed the data. JX wrote the manuscript. QH and FJ discussed the results and offered good suggestions. All the authors reviewed the manuscript.

## Conflict of Interest Statement

The authors declare that the research was conducted in the absence of any commercial or financial relationships that could be construed as a potential conflict of interest.

## Footnotes

^1^http://www.fil.ion.ucl.ac.uk/spm/.

^2^http://surfer.nmr.mgh.harvard.edu/.

^3^http://www.osirix-viewer.com/download_form/download_form.php.

^4^https://surfer.nmr.mgh.harvard.edu/fswiki/FreeviewGuide/FreeviewWorkingWithData/FreeviewEditingaRecon.
